# Small non‐coding RNAs are altered by short‐term sprint interval training in men

**DOI:** 10.14814/phy2.13653

**Published:** 2018-04-02

**Authors:** Joshua Denham, Adrian J. Gray, John Scott‐Hamilton, Amanda D. Hagstrom, Aron J. Murphy

**Affiliations:** ^1^ School of Science and Technology University of New England Armidale New South Wales Australia; ^2^ School of Health University of New England Armidale New South Wales Australia; ^3^ School of Environmental and Rural Science University of New England Armidale New South Wales Australia

**Keywords:** Epigenetics, exercise, HIIT, microRNA, miRNome

## Abstract

Small non‐coding RNAs (ncRNAs) are emerging as important molecules for normal biological processes and are deregulated in disease. Exercise training is a powerful therapeutic strategy that prevents cardiometabolic disease and improves cardiorespiratory fitness and performance. Despite the known systemic health benefits of exercise training, the underlying molecular mechanisms are incompletely understood. Recent evidence suggests a role for epigenetic mechanisms, such as microRNAs, but whether other small ncRNAs are modulated by chronic exercise training is unknown. Here, we used small RNA sequencing to explore whether sprint interval training (SIT) controls the abundance of circulating small ncRNAs in human whole blood samples. Ten healthy men performed SIT three times a week for 6 weeks. After training, subjects showed marked improvements in maximal oxygen consumption and cycling performance with concurrent changes to the abundance of diverse species of circulating small ncRNAs (*n *=* *1266 small ncRNAs, *n *=* *13 microRNAs, *q *<* *0.05). Twelve microRNAs altered by 6 weeks of SIT were ubiquitously expressed microRNAs and two regulated important signaling pathways, including p53, thyroid hormone and cell cycle signaling. MicroRNAs altered by 6 weeks of SIT were unchanged after a single session of SIT (*n *=* *24, all *P *>* *0.05). Relative to older individuals, younger subjects exhibited an increased acute SIT‐induced fold change in miR‐1301‐3p (*P *=* *0.02) – a microRNA predicted to target mRNAs involved in alternative splicing, phosphoprotein and chromosomal rearrangement processes (all *P *<* *0.001). Our findings indicate many species of circulating small ncRNAs are modulated by exercise training and that they could control signaling pathways responsible for health benefits achieved from exercise.

## Introduction

Exercise training is a powerful lifestyle factor that prevents, controls the symptoms, and regresses the severity of many age‐related diseases. Despite the known systemic health benefits elicited by routine exercise, the precise signaling pathways are poorly understood. The adaptations to exercise training involve genetic variants and recent evidence has implicated the role of epigenetic modifications (Denham 2017). These modifications include DNA methylation, histone protein modifications, and the regulation of microRNAs (miRNAs) (Denham 2017). The latter are a particular species of small, non‐coding RNAs (ncRNAs) that control gene expression through post‐transcriptional mechanisms or orchestrating conformational changes to chromatin.

Small ncRNAs are crucial for normal biological processes and become aberrantly expressed in diseases, such as cancer and atherosclerosis (Esteller 2011; Martens‐Uzunova et al. 2013). There are many species of small RNAs, though their precise roles are incompletely understood. However, some are involved in RNA processing, hypoxic pathways, apoptosis, immune‐regulation, and transcriptional programming (Martens‐Uzunova et al. 2013; Raina and Ibba 2014; Atianand et al. 2016). Circulating small ncRNAs, such as miRNAs, are responsible for intercellular signaling (Turchinovich et al. 2013; Guay and Regazzi 2017) and could control the holistic health benefits conferred by exercise training, as they are stably transported through the vasculature. For these reasons, miRNAs are proposed biomarkers of diseases and small RNA‐based therapies are showing promise in clinical settings (Barata et al. 2016; Byron et al. 2016). Many miRNAs are implicated in various exercise‐induced adaptations (e.g., physiological cardiac hypertrophy, mitochondrial biogenesis, insulin sensitivity) (Polakovicova et al. 2016; Denham 2017), yet whether chronic exercise training influences other species of small ncRNAs is currently unknown.

Recently, the acute exercise‐induced changes to small RNA molecules isolated from human plasma were revealed (Shah et al. 2017). Our study aimed to extend previous findings by: (1) characterizing the circulating small RNA changes to an effective form of short‐term, vigorous exercise training – sprint interval training (SIT) – and measuring them in context with the improvement to cardiorespiratory fitness and cycling performance; (2) determining the effect of short‐term SIT on the circulating miRNome; and (3) establishing whether miRNAs differentially regulated by chronic SIT were modulated by a single session of SIT.

## Materials and Methods

### Participants

The study procedures and training methods have been outlined elsewhere (Denham et al. 2017a,b). For the 6‐week training study, ten untrained individuals were recruited and completed SIT, three times a week on cycle ergometers. The apparently healthy men were recreationally active but were not currently engaged in any structured aerobic exercise training. Subjects completed 4–6 maximal efforts with 4 min recovery between sets. Subject characteristics are outlined in Table 1. Fasted blood samples were obtained before and 2–4 days after their final SIT session.

**Table 1:** Participant characteristics before and after 6 weeks of sprint interval training

	Before	After	Percent change (%)	*P*‐value
Sex (M)	*N *=* *10			
Age (y)	33.3 ± 10.9			
Height (cm)	183.4 ± 6.9			
Weight (kg)	90.2 ± 13.5	90.9 ± 12.4	0.95 ± 2.79	0.43
BMI (Weight/height^2^)	26.8 ± 3.5	26.8 ± 2.8	0.19 ± 4.23	0.93
$\dot{V}$O_2max_ (L^ ^min^−1^)	3.84 ± 0.72	4.20 ± 0.67	9.41 ± 6.66	0.001
$\dot{V}$O_2max_ (mL^ ^kg^−1 ^min^−1^)	42.8 ± 6.2	46.2 ± 6.0	8.45 ± 7.05	0.006
*P*_max_ (W)	299 ± 46.3	334 ± 47.9	12.06 ± 6.12	<0.001
*P*_max_ (W^ ^kg^−1^)	3.33 ± 0.37	3.70 ± 0.42	11.11 ± 7.23	0.001
FTP (W)	197.2 ± 42.0	223.2 ± 40.9	14.15 ± 8.14	<0.001
FTP (W^ ^kg^−1^)	2.19 ± 0.38	2.46 ± 0.33	13.13 ± 8.12	<0.001
*P*_mean_ (W)	616.3 ± 83.6	653 ± 95.9	6.76 ± 15.61	0.23
*P*_mean_ (W^ ^kg^−1^)	6.88 ± 0.72	7.25 ± 1.10	5.54 ± 13.38	0.22
*P*_peak_ (W)	935.7 ± 231.5	1026.4 ± 218.1	13.01 ± 26.65	0.16
*P*_peak_ (W^ ^kg^−1^)	10.36 ± 2.04	11.33 ± 2.12	11.60 ± 24.05	0.18

Descriptive data are expressed as mean ± SD.

Legend: M, male; BMI, body mass index; $\dot{V}$O_2max_, maximal oxygen consumption; *P*_max_, maximum power output during incremental testing; FTP, function threshold power (estimated from average power output obtained after a 20‐min test); *P*_mean_, average power output during 30‐sec Wingate test; *P*_peak_, peak power output during 30‐sec Wingate test.

For the acute trial, 24 individuals (mean ± SD: age, 35.7 ± 11.75 years; height, 179.9 ± 8.0 cm; weight, 81.4 ±12.0 kg; relative $\dot{V}$O_2max_, 46.86 ± 8.55 mL^ ^kg^−1^ min^−1^) completed a single session of SIT, comprised of four maximal 30‐sec sprints on a cycle ergometer as described previously (Denham et al. 2017a). These subjects donated a fasted blood sample before and 30 min after their fourth sprint effort. All subjects were apparently healthy according to the Exercise and Sports Science Australia (ESSA) prescreening tool and medical questionnaires. All subjects gave written informed consent and the study was approved by the University of New England's Human Research Ethics Committee (Approval number: HE15‐294).

### Small RNA sequencing

Total RNA was extracted from whole blood samples using the miRVana miRNA Mini Kit (Thermofisher Scientific, Australia). One mL of blood was treated with 5 volumes of the erythrocyte lysis buffer (buffer EL, Qiagen, Australia) and spun at 2700 rpm for 10 min. Whole blood cells were pelleted, resuspended in 2 volumes of buffer EL and centrifuged a second time. RNA was then extracted using the miRVana miRNA Mini Kit (Thermofisher Scientific, Australia), following the manufacturer's guidelines. The analysis of whole blood RNA enabled the quantitation of small RNAs in the extracellular fluid, erythrocytes and leukocytes.

Small RNA was sequenced with the assistance of the Australian Genome Research Facility (AGRF), Melbourne, Australia. As a quality control (QC), RNA purity and integrity were analyzed using the RNA 6000 Pico and the Bioanalyzer small RNA kits. All samples passed QC and demonstrated excellent purity with average (±SD) RNA integrity numbers (RIN) and rRNA ratios of 9.89 ± 0.25 and 1.53 ± 0.33, respectively). The NEB's NEBNext Multiplex Small RNA Library Pre‐Set was used to prepare small RNA libraries to target mature miRNAs and other small RNAs that possess a 3′OH group. After 3′ and 5′ adapter ligation and reverse transcription primer annealing, cDNA was synthesized, PCR amplified with indexed primers and purified from gels using the Pippin Prep system. Library size was assessed via electrophoresis using the Agilent TapeStation TapeScreen DNA 1000 Assay and quantified by quantitative PCR (qPCR) using the KAPA Library Quantification Kits (Illumina). The libraries were normalized to 2 nmol L^‐1^ and pooled for sequencing on the HiSeq 2500 with 50 bp single reads. Per base sequence quality for the 20 samples (*n *=* *10 before and after SIT) was excellent with >96% bases above Q30 across all samples. Reads were screened for the presence of any Illumina and NEB adapter or overrepresented sequences and cross‐species contamination, as per the AGRF QC measures. Sequence adapters were cleaned and reads were retained for processing. Similar reads were collapsed and their counts were recorded.

### Bioinformatics

The raw RNA sequencing counts were imported into R (version 3.4.0) and Bioconductor packages (Huber et al. 2015) were used to perform sample diagnostics and the differential expression analysis (Ritchie et al. 2015). Small RNA annotation was undertaken using the Unitas software (Gebert et al. 2017). Normalization factors were calculated to scale the raw library sizes and a common negative binomial dispersion parameter estimated using the Bioconductor package edgeR (Robinson et al. 2010; McCarthy et al. 2012) and normalized using RUVSeq (Risso et al. 2014), which reduces the variability in an unbiased manner by estimating variation by residuals. MiRNA target pathways were identified using the miRPathDB (Backes et al. 2017). The miRNA expression differences between paired samples were presented as log fold change. A false discovery rate (FDR) was applied to prevent inflated type 1 error – *P*‐values were Benjamini‐Hotchberg corrected to generate *q*‐values.

### MicroRNA validation by quantitative PCR

Approximately, 100 ng of RNA was reverse transcribed using the MicroRNA Reverse Transcription Kit (ThermoFisher Scientific, Australia) with miRNA‐specific TaqMan assays for hsa‐miR‐1301‐3p, hsa‐miR‐769‐5p, hsa‐miR‐423‐5p, hsa‐miR‐451a, hsa‐miR‐493‐3p, hsa‐miR‐370‐3p, and U6 snRNA (Assay ID: 002827, 001998, 002340, 001141, 002364, 002275 and 001973, respectively). Experimental procedures and thermos‐cycling conditions were described previously (Denham et al. 2017a). Relative miRNA abundance was calculated using the 2^‐delta‐delta^ Ct method and expressed as fold change. All intra‐assay coefficient of variations for miRNAs were, on average, <1%.

### Statistical analyses

Subject characteristics and small ncRNA abundance before and after the SIT intervention (two‐tailed paired samples *t*‐tests or Wilcoxon signed‐rank tests) were assessed using IBM SPSS Statistics for Mac (IBM Corp., USA). Two‐tailed independent samples *t*‐tests were used to explore differences in individuals with increases in miRNAs after acute SIT compared to those with decreases. Statistical significance was set at *P *<* *0.05.

## Results

### Sprint interval training improves cardiorespiratory fitness and cycling performance

Subjects’ relative $\dot{V}$O_2max_ (mL^ ^kg^−1 ^min^−1^), *P*_max_ (W^ ^kg^−1^), and functional threshold power (W^ ^kg^−1^) were all improved after 6 weeks of SIT (mean ± SD percent change: 8.45 ± 7.0, 11.11 ± 7.2, and 13.13 ± 8.1, respectively, all *P *≤* *0.001). A post hoc power analysis revealed that we achieved >80% power to detect the observed changes to relative $\dot{V}$ O_2max_ (mL^ ^kg^−1 ^min^−1^), *P*_max_ (W^ ^kg^−1^) and functional threshold power (W^ ^kg^−1^) after SIT. Body weight and body mass index were unchanged after SIT and despite increases in 30‐sec mean power (*P*_mean_) and 30‐sec peak power (*P*_peak_), they were not statistically significant (both *P *>* *0.05, Table 1).

### Sprint interval training regulates circulating small RNAs

**Figure 1:**
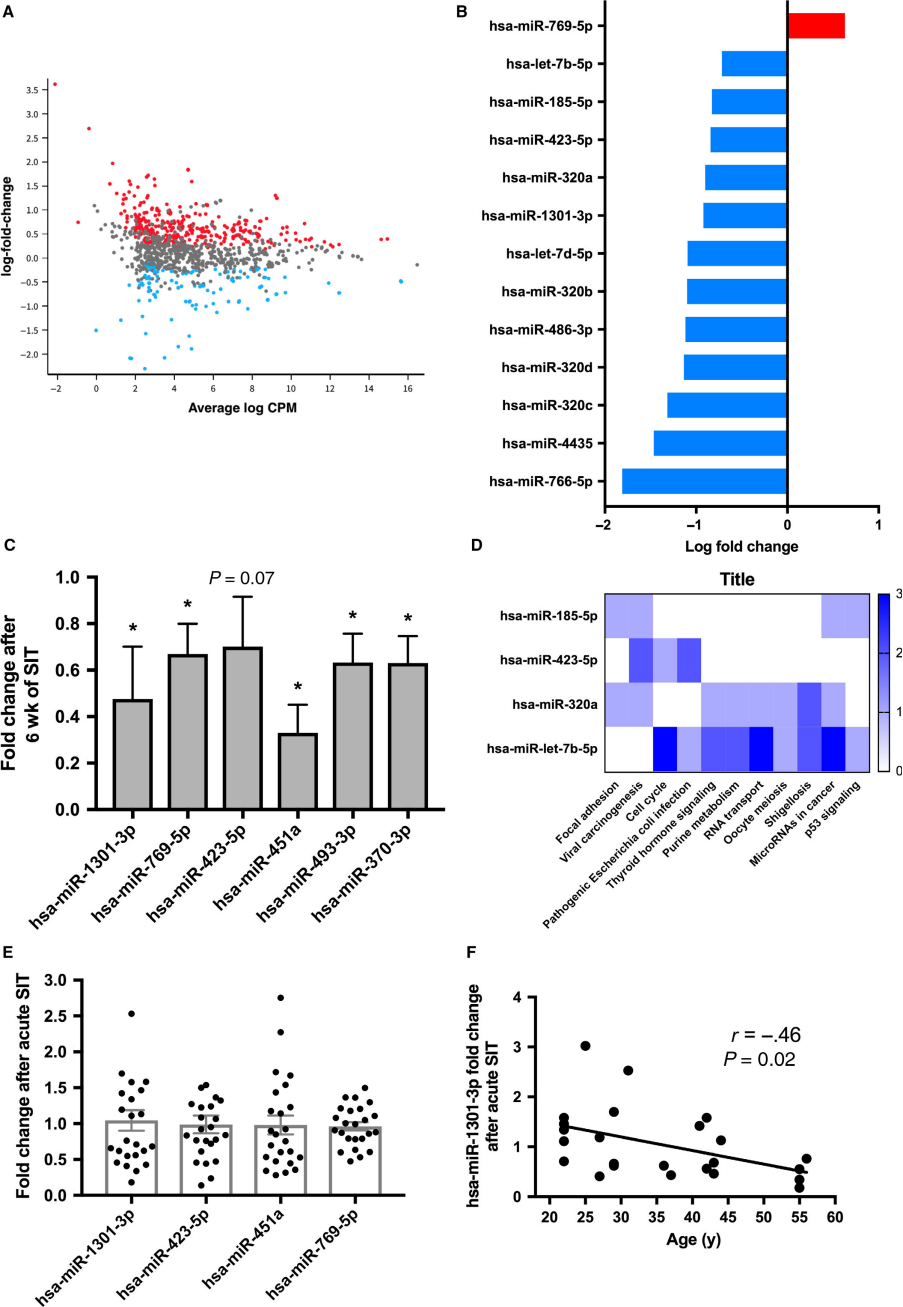
Small non‐coding RNA changes after sprint interval training in humans. (A) A scatterplot of small non‐coding RNA (ncRNA) changes after 6 weeks of sprint interval training (SIT) in 10 healthy men. The average log counts per million (CPM) and log fold change are on the x and y axis, respectively. While red indicates small ncRNAs increased after SIT, those decreased are displayed in blue (*n *=* *1266, *q *<* *0.05). (B) MicroRNA changes after 6 weeks of sprint interval training (SIT). After applying a false discovery rate correction, the abundance of 13 miRNAs were altered by SIT (*n *=* *10, *q *<* *0.05). (C) MicroRNA abundance validation by quantitative PCR. Six miRNAs that were modulated by SIT were experimentally validated by qPCR. All miRNAs were significantly decreased after SIT (*n *=* *8–10, all *P *≤* *0.05), except for hsa‐miR‐423‐5p which approached statistical significance (*P *=* *0.07). Data are from Wilcoxon signed‐rank tests and are displayed as mean ± SEM. (D) MicroRNA pathway analysis. Pathways modulated by miRNAs were identified using the miRPathDB (Backes et al. 2017). A heat map of pathways targeted by at least two miRNAs (of the 13 influenced by SIT,* q *<* *0.05), as indicated by experimental evidence are displayed. (E) MicroRNA changes 30 min after a single session of SIT. No consistent changes were observed in the four miRNAs after acute SIT (*n *=* *24, all *P *>* *0.05). (F) Linear relationship between age and the acute SIT‐induced change to miR‐1301‐3p (*n *=* *24, *r *=* *−0.46, *P *=* *0.02). Individuals with a decrease in miR‐1301‐3p after SIT were, on average, 11 y older than those with an increase after acute SIT (40.7 ± 11.9 years vs. 29.7 ± 8.7 years, respectively, *P *=* *0.02). Data were from Pearson's correlation and independent samples *t*‐tests. *P ≤* *0.05.

All RNA samples passed QC and were successfully sequenced. A number of 2066 small ncRNAs were detected in whole blood samples of healthy men (*n *=* *10). Of these, 1266 were influenced by 6 weeks of SIT (*q *<* *0.05, Fig. 1A). Four hundred and thirty‐two (log FC:.19–3.62) and 834 were up and downregulated (−0.9 to −2.30), respectively (*q *<* *0.05). The most statistically significant small ncRNAs altered by SIT are presented in Table 2 (*q *<* *0.01). Table 3 displays the small ncRNAs with the largest magnitude of change after 6 weeks of SIT (*q *<* *0.05).

**Table 2:** Small non‐coding RNAs modulated by 6 weeks of sprint interval training (*q *<* *0.01)

Small non‐coding RNA	Log FC	*P*‐value	*q*‐value
SGMS1‐AS1‐ENSG00000226200.6	2.69	0.0003	0.008
tRNA‐Phe‐GAA‐10‐1	1.72	0.00003	0.003
tRNA‐Glu‐CTC‐5‐1	1.68	0.00002	0.003
BX005132.1‐ENSG00000284674.1	1.60	0.00025	0.007
LINC01249‐ENSG00000231532.5	1.54	0.0001	0.006
tRNA‐Glu‐TTC‐10‐1	1.48	0.00004	0.005
GENSCAN00000046530	1.37	0.0001	0.006
tRNA‐Asp‐GTC‐5‐1	1.30	0.00001	0.003
tRNA‐Asp‐GTC‐4‐1	1.26	0.00000	0.00065
tRNA‐Val‐TAC‐chr2‐6	1.16	0.0002	0.007
Y_RNA‐ENSG00000222224.1	1.15	0.0003	0.008
GENSCAN00000027729	1.13	0.00003	0.003
GENSCAN00000017883	1.12	0.0002	0.007
AC062029.1‐(2)	1.10	0.00008	0.006
GENSCAN00000039883	1.03	0.00017	0.007
CASC2‐(3)	1.00	0.0002	0.007
GENSCAN00000017737	0.99	0.0001	0.006
GENSCAN00000037186	0.98	0.0001	0.006
GENSCAN00000006535	0.98	0.00005	0.005
GENSCAN00000007667	0.95	0.00002	0.003
GENSCAN00000025648	0.95	0.00002	0.003
GENSCAN00000026399	0.94	0.00002	0.003
GENSCAN00000023054	0.93	0.0003	0.008
Y_RNA‐ENSG00000201118.1	0.92	0.0001	0.006
GENSCAN00000037532	0.90	0.00001	0.003
GENSCAN00000014910	0.90	0.0003	0.007
GENSCAN00000006901	0.89	0.0003	0.008
tRNA‐Lys‐TTT‐6‐1	0.88	0.0004	0.009
tRNA‐Lys‐TTT‐4‐1	0.86	0.0003	0.008
tRNA‐Gly‐CCC‐chr1‐16	0.83	0.00000	0.0025
tRNA‐Gly‐CCC‐chr1‐19	0.83	0.00000	0.0025
tRNA‐Pro‐TGG‐2‐1	0.82	0.0002	0.007
Y_RNA‐ENSG00000201196.1	0.81	0.0001	0.006
GENSCAN00000044961	0.80	0.0001	0.006
RNY4P19‐ENSG00000199400.1	0.77	0.0002	0.007
tRNA‐Arg‐TCG‐4‐1	0.77	0.00001	0.003
tRNA‐Arg‐TCG‐5‐1	0.77	0.00001	0.003
tRNA‐Thr‐TGT‐5‐1	0.76	0.0003	0.007
GENSCAN00000025006	0.75	0.0001	0.006
tRNA‐Arg‐TCG‐2‐1	0.75	0.00003	0.003
tRNA‐Thr‐TGT‐2‐1	0.75	0.0003	0.008
tRNA‐Thr‐TGT‐3‐1	0.73	0.0004	0.009
tRNA‐Arg‐TCG‐1‐1	0.73	0.0003	0.008
tRNA‐Thr‐CGT‐1‐1	0.72	0.0001	0.006
tRNA‐Phe‐GAA‐3‐1	0.70	0.00002	0.003
tRNA‐Thr‐AGT‐3‐1	0.70	0.0002	0.007
tRNA‐Thr‐TGT‐1‐1	0.69	0.0002	0.007
tRNA‐Phe‐GAA‐5‐1	0.69	0.00001	0.003
tRNA‐Asp‐GTC‐3‐1	0.69	0.00001	0.003
tRNA‐Thr‐AGT‐1‐1	0.66	0.0003	0.008
tRNA‐Thr‐AGT‐1‐2	0.66	0.0003	0.008
tRNA‐Thr‐AGT‐1‐3	0.66	0.0003	0.008
tRNA‐Phe‐GAA‐2‐1	0.63	0.00008	0.006
GENSCAN00000043979	0.63	0.00025	0.007
AC004494.1‐ENSG00000262312.2	0.63	0.00029	0.008
GENSCAN00000035656	0.62	0.00006	0.005
AC084082.1‐(2)	0.62	0.0002	0.007
Y_RNA‐ENSG00000238585.1	0.62	0.0004	0.009
GENSCAN00000048902	0.62	0.00005	0.005
tRNA‐Phe‐GAA‐1‐1	0.61	0.0001	0.006
tRNA‐Phe‐GAA‐1‐2	0.61	0.0001	0.006
tRNA‐Phe‐GAA‐1‐3	0.61	0.0001	0.006
tRNA‐Phe‐GAA‐1‐4	0.61	0.0001	0.006
tRNA‐Phe‐GAA‐1‐5	0.61	0.0001	0.006
tRNA‐Phe‐GAA‐1‐6	0.61	0.0001	0.006
tRNA‐Gly‐CCC‐5‐1	0.60	0.0002	0.007
Y_RNA‐ENSG00000199949.2	0.59	0.00002	0.003
Y_RNA‐ENSG00000207207.1	0.59	0.0003	0.007
Y_RNA‐ENSG00000201548.1	0.59	0.0002	0.006
Y_RNA‐ENSG00000222881.1	0.57	0.0004	0.01
MT‐TH‐ENSG00000210176.1	0.56	0.0004	0.008
Y_RNA‐ENSG00000207271.1	0.56	0.00045	0.001
Y_RNA‐ENSG00000199366.1	0.53	0.0001	0.006
Y_RNA‐ENSG00000201933.1	0.52	0.0003	0.007
SNORD51‐ENSG00000207047.2	0.50	0.0002	0.006
SNORD51‐ENSG00000283671.1	0.50	0.0002	0.006
GENSCAN00000032595	0.45	0.0004	0.0085
RMRP‐ENSG00000277027.1	0.29	0.0002	0.007
SSU:LCYE01007731.2122.3984	−0.27	0.0003	0.007
SSU:LKHY01001625.8471.10331	−0.36	0.0004	0.01
RN7SL2‐ENSG00000274012.1	−0.59	0.0004	0.0085
tRNA‐Ser‐CGA‐1‐1	−0.62	0.0003	0.007
tRNA‐Ser‐CGA‐2‐1	−0.62	0.0003	0.007
SNORA62‐ENSG00000202363.1	−0.91	0.00007	0.006
SNORD41‐ENSG00000209702.1	−1.06	0.00011	0.006
Y_RNA‐ENSG00000202144.1	−1.57	0.00009	0.006
Y_RNA‐ENSG00000200090.1	−1.84	0.0003	0.007
Y_RNA‐ENSG00000199410.1	−2.08	0.00007	0.006
Y_RNA‐ENSG00000200834.1	−2.08	0.0002	0.007
Y_RNA‐ENSG00000201555.1	−2.09	0.0001	0.006
Y_RNA‐ENSG00000264393.1	−2.09	0.0001	0.006
Y_RNA‐ENSG00000264778.1	−2.09	0.0001	0.006
Y_RNA‐ENSG00000264887.1	−2.09	0.0001	0.006
Y_RNA‐ENSG00000264955.1	−2.09	0.0001	0.006
Y_RNA‐ENSG00000266113.1	−2.09	0.0001	0.006
Y_RNA‐ENSG00000199580.1	−2.30	0.00005	0.005

Legend: FC, fold change.

**Table 3:** Small non‐coding RNAs modulated by 6 weeks of sprint interval training (*q *<* *0.05)

Small non‐coding RNA (ID)	Log FC	*P*‐value	*q*‐value
LINC01237‐ENSG00000233806.7	3.62	0.005	0.01
GENSCAN00000033485	1.97	0.002	0.01
tRNA‐Tyr‐GTA‐chr1‐127	1.84	0.001	0.01
tRNA‐Tyr‐GTA‐chr21‐2	1.84	0.001	0.01
tRNA‐Tyr‐GTA‐chr14‐8	1.84	0.001	0.01
tRF‐1_tRNA‐Val‐TAC‐1‐2	1.64	0.0005	0.01
MT‐TY‐ENSG00000210144.1	1.59	0.001	0.01
tRF‐1_[3]_tRNA‐Asp‐GTC‐2‐3	1.53	0.006	0.01
SSU:LRIL01008447.2849.4725	1.51	0.02	0.03
tRNA‐leader_[3]_tRNA‐Ser‐GCT‐4‐2	1.34	0.004	0.01
GENSCAN00000032631	1.32	0.02	0.03
tRNA‐Lys‐CTT‐4‐1	1.30	0.01	0.02
tRNA‐Gly‐CCC‐7‐1	1.29	0.002	0.01
tRNA‐Lys‐CTT‐1‐1	1.24	0.01	0.02
tRNA‐Lys‐CTT‐1‐2	1.24	0.0108356	0.02
tRF‐1_tRNA‐Val‐CAC‐1‐2	1.24	0.0006	0.01
tRF‐1_tRNA‐Asp‐GTC‐1‐1	1.22	0.0025	0.01
GENSCAN00000025626	1.13	0.02	0.04
tRNA‐Lys‐TTT‐5‐1	1.11	0.004	0.01
tRNA‐Lys‐TTT‐3‐1	1.10	0.00	0.01
tRNA‐Lys‐TTT‐3‐2	1.10	0.004	0.01
tRNA‐Lys‐TTT‐3‐3	1.10	0.004	0.01
tRNA‐Lys‐TTT‐3‐4	1.10	0.004	0.01
tRNA‐Lys‐TTT‐3‐5	1.10	0.004	0.01
tRNA‐leader_[6]_tRNA‐His‐GTG‐1‐5	1.06	0.0095	0.02
tRF‐1_tRNA‐Arg‐CCT‐3‐1	1.04	0.02	0.04
tRF‐1_tRNA‐Thr‐CGT‐2‐1	1.04	0.001	0.01
tRF‐1_tRNA‐Thr‐TGT‐4‐1	1.01	0.03	0.04
tRNA‐Glu‐TTC‐6‐1	1.00	0.002	0.01
tRF‐1_[6]_tRNA‐Phe‐GAA‐1‐4	0.98	0.0008	0.01
GENSCAN00000007596	0.95	0.0008	0.01
SNORD31B‐ENSG00000201847.1	0.94	0.005	0.01
GENSCAN00000017537	0.94	0.002	0.01
GENSCAN00000003559	0.92	0.02	0.04
AC124068.2‐ENSG00000261441.1	0.92	0.02	0.035
GENSCAN00000045642	0.90	0.009	0.02
TEX41‐(59)	0.90	0.02	0.04
tRNA‐leader_tRNA‐His‐GTG‐1‐5	0.87	0.003	0.01
GENSCAN00000037392	0.86	0.004	0.01
GENSCAN00000012181	0.86	0.005	0.01
GENSCAN00000033127	0.85	0.006	0.01
tRNA‐Ala‐AGC‐19‐1	0.85	0.002	0.01
tRNA‐Ala‐AGC‐21‐1	0.85	0.002	0.01
GENSCAN00000040198	0.83	0.02	0.035
GENSCAN00000035617	0.82	0.01	0.02
RNU2‐37P‐ENSG00000222627.1	−0.81	0.008	0.015
tRNA‐Val‐AAC‐1‐1	−0.87	0.009	0.02
tRNA‐Val‐AAC‐1‐2	−0.87	0.009	0.02
tRNA‐Val‐AAC‐1‐3	−0.87	0.009	0.02
tRNA‐Val‐AAC‐1‐4	−0.87	0.009	0.02
tRNA‐Val‐AAC‐1‐5	−0.87	0.009	0.02
tRNA‐Val‐AAC‐3‐1	−0.87	0.009	0.02
tRNA‐Val‐AAC‐4‐1	−0.87	0.009	0.02
SNORD23‐ENSG00000221803.1	−0.88	0.006	0.01
tRNA‐Val‐AAC‐6‐1	−0.89	0.0007	0.01
tRNA‐Ala‐AGC‐2‐1	−0.89	0.00075	0.01
tRNA‐Ala‐AGC‐2‐2	−0.89	0.00075	0.01
tRNA‐Ala‐AGC‐3‐1	−0.90	0.00075	0.01
SNHG20‐(5)	−0.90	0.005	0.01
tRNA‐Ala‐CGC‐4‐1	−0.90	0.0008	0.01
tRNA‐Val‐CAC‐2‐1	−0.91	0.002	0.01
SCARNA16‐ENSG00000275143.1	−0.91	0.005	0.01
tRNA‐Ala‐AGC‐7‐1	−0.91	0.0007	0.01
tRNA‐Ala‐AGC‐5‐1	−0.92	0.0007	0.01
RNY3‐ENSG00000202354.1	−0.95	0.003	0.01
SNORD35B‐ENSG00000200530.1	−0.97	0.001	0.01
SCARNA7‐ENSG00000238741.1	−0.99	0.0009	0.01
Y_RNA‐ENSG00000202273.1	−1.01	0.0007	0.01
SNORD83B‐ENSG00000209480.1	−1.06	0.0005	0.01
SNORD89‐ENSG00000212283.1	−1.13	0.004	0.01
LINC02001‐ENSG00000267321.2	−1.22	0.002	0.01
SSU:LCYE01003164.11174.12956	−1.28	0.003	0.01
RNA5SP204‐ENSG00000201415.1	−1.29	0.0025	0.01
GENSCAN00000024975	−1.51	0.02	0.03
SNORD67‐ENSG00000212135.1	−1.62	0.0015	0.01
Y_RNA‐ENSG00000200291.1	−1.89	0.0009	0.01

Small ncRNAs with a fold change equal or less than −0.85 and equal or more than 0.85 after 6 weeks of SIT. Legend: FC, fold change.

### MicroRNA changes after 6 weeks of sprint interval training

Of the 211 miRNAs altered by SIT, 77 were increased (log fold change: .18–2.26) and 134 were decreased (−0.35 to −2.82, all *P *<* *0.05). After applying an FDR, 13 miRNAs were significantly altered by SIT (all *q *<* *0.05, Fig. 1B). Of these, one increased and 12 decreased after SIT (Fig. 1B). According to the miRmine database, 12 of the 13 miRNAs altered by SIT were ubiquitously expressed among 15 different tissues (range: 8–15) (Panwar et al. 2017). MiR‐4435 was only detectable in plasma (Panwar et al. 2017). Six miRNAs that were statistically significant or borderline statistically significant after FDR that had mRNA targets implicated in exercise signaling pathways were technically validated by qPCR. Quantitative PCR confirmed that miR‐1301‐3p, miR‐769‐5p, and miR‐451a were altered after 6 weeks of SIT (all *P* ≤ 0.05 Fig. 1C), and miR‐423‐5p approached borderline statistical significance (*P *=* *0.07). We also found miR‐493‐3p and miR‐370‐3p were decreased after SIT (both *P *≤ 0.05, Fig. 1C). Using the miRPathDB database (Backes et al. 2017), an analysis of miRNAs implicated in biological and disease pathways was performed on the 13 miRNAs altered after SIT (*q *<* *0.05). Experimental evidence suggested that two or more miRNAs were implicated in diverse pathways including cell cycle, thyroid hormone‐ and p53 signaling, and miRNAs in cancer (Fig. 1D).

### MicroRNA abundance after acute sprint interval training

No consistent miRNA changes were observed 30‐min after acute SIT, due to the large interindividual variation in responses (*n *=* *24, all *P *>* *0.05, Fig. 1E). The fold change in miR‐1301‐3p after acute SIT was negatively correlated to age (*r *=* *−0.45, *P *=* *0.03, Fig. 1F). Those with a decrease (FC < 1.0) in miR‐1301‐3p after SIT were younger compared to those with increased (FC > 1.0) miR‐1301‐3p (*n *=* *13, 40.69 ± 11.93 years vs. *n *=* *11, 29.73 ± 8.67 years, *P *=* *0.02). No other statistically significant differences were observed between those who decreased versus those who increased miR‐1301‐3p after acute SIT (all *P *>* *0.05). Using the miRWalk database, we identified predicted mRNA targets of miR‐1301‐3p and submitted them to DAVID for functional annotation analysis. The predicted mRNA targets of miR‐1301‐3p were enriched for alternative splicing, phosphoprotein, and chromosomal rearrangement processes (all *P *<* *0.001).

## Discussion

Small ncRNAs have emerged as modulators of gene expression through controlling epigenetic modifications and interactions with mRNAs (splicing, processing, and destabilizing). Specific non‐coding RNAs, such as miRNAs, are implicated in exercise adaptations and circulating miRNAs could eventually serve as biomarkers of exercise adaptations and utilized for the development of personalized exercise prescription, or developed into novel RNA‐based therapies for the treatment of cardiometabolic disease (Kirby and McCarthy 2013; Polakovicova et al. 2016; Safdar et al. 2016; Denham 2017). Using small RNA sequencing and SIT as a potent form of exercise training to elicit significant improvements in $\dot{V}$O_2max_ and cycling performance, we performed the first comprehensive analysis of the circulating small non‐coding RNA changes to short‐term exercise training.

MicroRNAs in plasma (Nielsen et al. 2014; Van Craenenbroeck et al. 2015; Zhang et al. 2017), skeletal muscle (Rowlands et al. 2014) and whole blood (Hecksteden et al. 2016) are malleable to training interventions in various cohorts of healthy, athletic, or diseased humans. To date, only one investigation has assessed the whole blood miRNome response to a 6‐day training intervention in athletes (strength and endurance) (Hecksteden et al. 2016). The previous investigation explored miRNA changes to physical fatigue and did not analyze the miRNome changes in context with adaptations to exercise (Hecksteden et al. 2016). Moreover, it is likely that miRNome response to exercise would be different between athletic and untrained individuals. Whether other small ncRNAs are vital for controlling exercise adaptations is relatively unknown. Recently, the acute exercise‐induced changes to small ncRNAs in plasma were revealed (Shah et al. 2017). As the acute effects of exercise are typically proinflammatory and required for adaptation, the many health benefits gained from regular exercise occur over long‐term with training. Therefore, the purpose of our study was to explore the effect of a short‐term sprint interval training (SIT) intervention on small ncRNAs in whole blood samples from healthy, untrained men. Consistent with the effects of acute exercise (Shah et al. 2017), chronic exercise training resulted in marked changes to circulating small ncRNA abundance in a diverse RNA species.

Although only 13 miRNAs were regulated by SIT after applying an FDR (*q *<* *0.05), many ncRNAs were differentially expressed (*n *=* *1266, *q *<* *0.05). The precise biological roles of each species of small ncRNAs are beginning to be elucidated. Small ncRNAs, however, seem to regulate gene expression and protein content through various mechanisms. For instance, lincRNA‐EPS is an immunoregulatory lincRNA that negatively regulates the inflammatory responses in immune cells through interactions with chromatin and nucleosome repositioning (Atianand et al. 2016). Mitochondrial transfer RNAs (mt tRNAs) are transcribed by mitochondrial DNA for the translation of mitochondrial proteins and mutations in mt tRNA genes are linked to mitochondrial dysfunction (Suzuki et al. 2011). Others function by influencing RNA processing, splicing, protein synthesis or are yet to be delineated (e.g., Miscellaneous RNAs [miscRNAs], ribosomal RNA [rRNA] and vault RNAs, small nucleolar RNAs [snoRNAs], transfer RNA‐derived fragments) (Martens‐Uzunova et al. 2013). Importantly, our novel findings of the diverse ncRNAs species modulated by chronic training in whole blood samples suggest small ncRNAs (e.g., antisense RNAs, tRNAs, lincRNAs, mt tRNAs, snoRNAs, miscRNAs, and tRFs) other than miRNAs may regulate exercise‐induced adaptations.

It has become clear that exercise is a powerful lifestyle strategy capable of reprogramming the epigenome through modifications to DNA methylation, histone proteins, and microRNAs (Denham 2017). We previously found four miRNAs important for muscle development were reduced in whole blood samples after 6 weeks of SIT (Denham et al. 2017a). Here, we extended our previous findings by using small RNA sequencing and revealed the global changes in diverse species of small ncRNAs isolated from whole blood after SIT, including 13 miRNAs after FDR‐correction. The miRNAs were not consistently regulated by acute exercise, possibly due to large interindividual responses. Most of the miRNA changes observed after SIT were ubiquitously expressed throughout the body, which could suggest systemic epigenetic reprogramming of miRNA genes. Indeed, miRNA genes are vulnerable to DNA methylation changes caused by SIT (Denham et al. 2015). Whether the systemic epigenetic reprogramming of tissue transcriptomes involves miRNAs and other small ncRNAs deserves attention.

Previous studies have found changes in specific circulating miRNAs after acute vigorous aerobic exercise (Radom‐Aizik et al. 2012; Cui et al. 2016; Kilian et al. 2016; Wahl et al. 2016) and longer exercise training interventions (Taurino et al. 2010; Van Craenenbroeck et al. 2015). Some miRNAs modulated after acute interval training in PBMCs were influenced by the short‐term vigorous exercise training in our study (e.g., miR‐320, miR‐486‐5p, let‐7 family members) (Radom‐Aizik et al. 2012). MiR‐486 is a muscle‐enriched miRNA that targets *PTEN* and controls the PI3K signaling pathway (Small et al. 2010). Endurance athletes possess higher whole blood miR‐486‐5p compared to healthy controls and miR‐486‐5p is correlated to $\dot{V}$O_2max_ (Denham and Prestes 2016). Here, miR‐486‐3p was the most statistically significantly reduced miRNA in individuals with $\dot{V}$O_2max_ improvements to SIT. Twelve weeks of endurance training altered the abundance of eight miRNAs isolated from plasma and a further four were borderline statistically significant after Bonferroni adjustment in seven individuals (Nielsen et al. 2014). Similar to the previous findings of miRNA changes in plasma after 12 weeks of endurance exercise (Nielsen et al. 2014), let‐7d, miR‐186 and miR‐766 were also reduced after 6 weeks of SIT in our study. Others that were decreased after short‐term SIT in our study were increased in plasma samples from individuals after acute maximal aerobic exercise (miR‐185‐5p, miR‐769‐5p and miR‐423‐3p) (Shah et al. 2017). Two of the three miRNAs (miR‐185‐5p and miR‐423‐3p) targeted pathways such as viral carcinogenesis, cell cycle, p53 signaling, and microRNAs in cancer. It is also interesting to note that some of the top miRNAs (miR‐423, miR‐320a, miR‐185, let‐7 family members) regulated by SIT are the most abundant miRNAs in human mitochondria and could control mitochondrial biogenesis and oxidative signaling pathways (Sripada et al. 2012). Thus, future work should explore how the exercise‐induced changes in circulating small ncRNAs regulate signaling pathways and what effects these have for disease prevention and exercise adaptations. Small ncRNAs could be responsible for fine‐tuning transcriptional profiles between tissues (Turchinovich et al. 2013; Guay and Regazzi 2017). While the fate of circulating miRNAs is unclear, they are stable and transportable in plasma. Therefore, it will be important to determine the biological functions of circulating miRNAs modulated by exercise, as they could eventually be developed into RNA‐based therapies to combat cardiometabolic disease.

There were some limitations to our study. We only included a modest number of men in the 6‐week SIT intervention (*n *=* *10). While the sample size raises concerns on statistical power, we used a conservative FDR‐correction on *P*‐values to provide *q*‐values and prevent the inflation of type 1 error. Larger studies could uncover more consistent changes in additional miRNAs and should investigate their responses in context with low‐ and high‐responders to particular exercise interventions. Diet and other environmental factors such as perceived stress, sleep patterns, and anxiety were not monitored throughout the intervention period and could also have influenced the findings, though we have no reason to expect such lifestyle perturbations. A control group was not included in our study and would be necessary to account for normal temporal effects on small ncRNAs in future exercise training interventions. Finally, we cannot account for the mechanisms leading to changes in small ncRNAs and their biological functions. While the ncRNAs could serve as a molecular signature to monitor exercise training and predict responses in humans, their utility as biomarkers requires further investigation.

In conclusion, we uncovered diverse changes to circulating small ncRNAs isolated from whole blood after a 6‐week SIT intervention. We provide the first evidence that chronic exercise modulates many species of circulating small ncRNAs in humans, emphasizing the profound effect exercise has epigenetic regulation of gene expression. The ubiquitously expressed miRNAs modulated by chronic SIT raise the possibility of systemic reprogramming caused by exercise. Although the roles of the circulating small ncRNAs are only beginning to be elucidated, our data suggest ncRNAs could underpin some of the adaptations to exercise training.

## Conflict of Interest

None declared.
